# Crystal structure of tolyl­fluanid

**DOI:** 10.1107/S1600536814020741

**Published:** 2014-09-20

**Authors:** Seonghwa Cho, Jineun Kim, Gihaeng Kang, Tae Ho Kim

**Affiliations:** a Department of Chemistry and Research Institute of Natural Sciences, Gyeongsang, National University, Jinju 660-701, Republic of Korea

**Keywords:** crystal structure, tolyl­fluanid, fungicide, hydrogen bonds

## Abstract

The title compound, C_10_H_13_Cl_2_FN_2_O_2_S_2_ {systematic name: *N*-[(di­chloro­fluoro­methyl)­sulfanyl]-*N′,N′*-dimethyl-*N*-*p*-tolyl­sulfamide}, is a well known fungicide. The dihedral angle between the mean plane of the di­methyl­amino group and that of the benzene ring is 32.3 (3)°. One Cl atom and one F atom of the di­chloro­fluoro­methyl­thio group are disordered over two sets of sites with an occupancy ratio of 0.605 (9):0.395 (9). In the crystal structure, two C—H⋯Cl hydrogen bonds link adjacent mol­ecules, forming dimers with *R*_2_^2^(14) loops. C—H⋯O hydrogen bonds link pairs of dimers into chains along the *b-*axis direction. These chains are joined by an additional C—H⋯O contact, generating a sheet in the *ab* plane.

## Related literature   

For information on the toxicity and fungicidal properties of the title compound, see: Sargis *et al.* (2012 ▶); Stajnbaher & Zupancic-Kralj (2008 ▶). For a related crystal structure, see: Ogata *et al.* (1986 ▶).
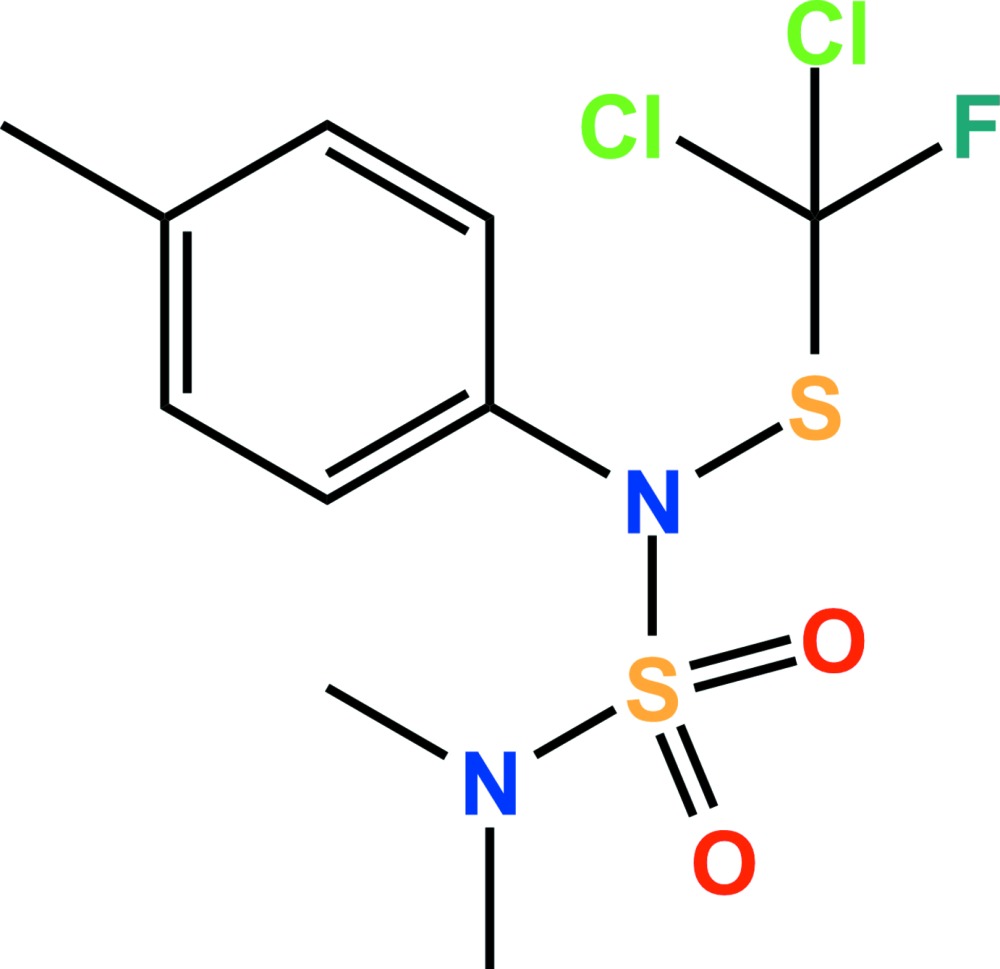


## Experimental   

### Crystal data  

C_10_H_13_Cl_2_FN_2_O_2_S_2_
*M* *_r_* = 347.24
Monoclinic, 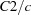

*a* = 23.7638 (19) Å
*b* = 8.7046 (7) Å
*c* = 14.6559 (11) Å
β = 102.133 (3)°
*V* = 2963.9 (4) Å^3^
*Z* = 8
Mo *K*α radiation
μ = 0.73 mm^−1^
*T* = 173 K
0.19 × 0.10 × 0.08 mm

### Data collection  

Bruker APEXII CCD diffractometer
Absorption correction: multi-scan (*SADABS*; Bruker, 2009 ▶) *T*_min_ = 0.874, *T*_max_ = 0.944
18246 measured reflections
2913 independent reflections
2236 reflections with *I* > 2σ(*I*)
*R*_int_ = 0.059

### Refinement  

*R*[*F* ^2^ > 2σ(*F* ^2^)] = 0.058
*wR*(*F* ^2^) = 0.135
*S* = 1.14
2913 reflections
194 parameters
H-atom parameters constrained
Δρ_max_ = 0.48 e Å^−3^
Δρ_min_ = −0.50 e Å^−3^

### 

Data collection: *APEX2* (Bruker, 2009 ▶); cell refinement: *SAINT* (Bruker, 2009 ▶); data reduction: *SAINT*; program(s) used to solve structure: *SHELXTL* (Sheldrick, 2008 ▶); program(s) used to refine structure: *SHELXTL*; molecular graphics: *DIAMOND* (Brandenburg, 2010 ▶); software used to prepare material for publication: *SHELXTL*.

## Supplementary Material

Crystal structure: contains datablock(s) global, I. DOI: 10.1107/S1600536814020741/sj5426sup1.cif

Structure factors: contains datablock(s) I. DOI: 10.1107/S1600536814020741/sj5426Isup2.hkl

**Fig. 1.**
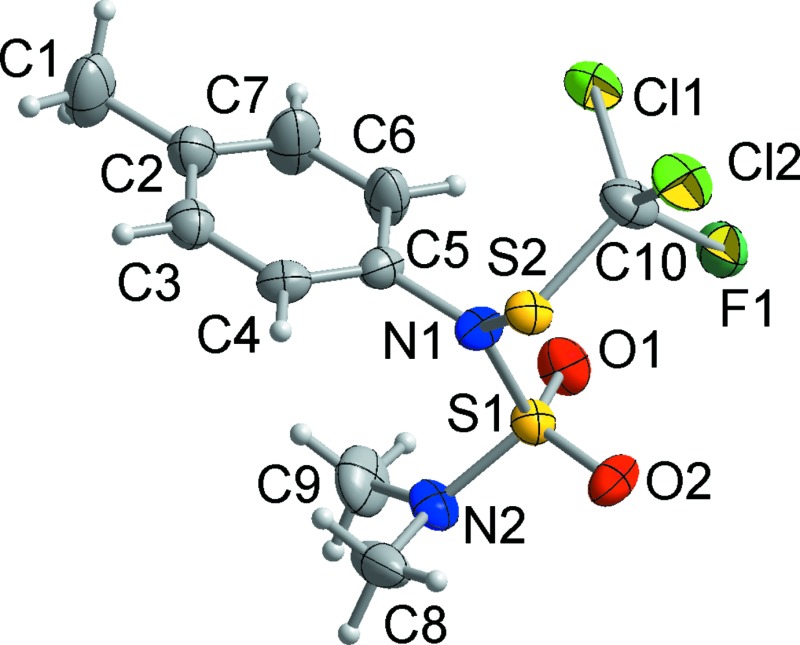
The asymmetric unit of the title compound with the atom numbering scheme. Displacement ellipsoids are drawn at the 50% probability level. H atoms are shown as small spheres of arbitrary radius. Only atoms of the major disorder components are shown.

. DOI: 10.1107/S1600536814020741/sj5426fig1.tif
The asymmetric unit of the title compound with the atom numbering scheme. Displacement ellipsoids are drawn at the 50% probability level. H atoms are shown as small spheres of arbitrary radius. Only atoms of the major disorder components are shown.

**Fig. 2.**
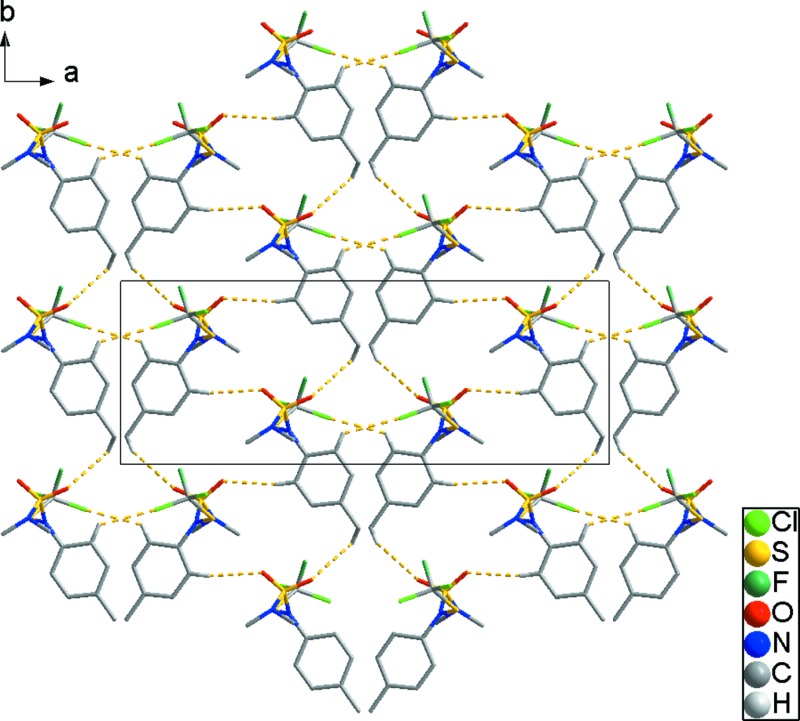
Crystal packing of the title compound with C–H···Cl and C–H···O hydrogen bonds are shown as dashed lines. H atoms bonded to C atoms have been omitted for clarity, except H atoms of hydrogen bonds. Only atoms of the major disorder components are shown.

. DOI: 10.1107/S1600536814020741/sj5426fig2.tif
Crystal packing of the title compound with C–H⋯Cl and C–H⋯O hydrogen bonds are shown as dashed lines. H atoms bonded to C atoms have been omitted for clarity, except H atoms of hydrogen bonds. Only atoms of the major disorder components are shown.

CCDC reference: 1024472

Additional supporting information:  crystallographic information; 3D view; checkCIF report

## Figures and Tables

**Table 1:** Hydrogen-bond geometry (Å, °)

*D*—H⋯*A*	*D*—H	H⋯*A*	*D*⋯*A*	*D*—H⋯*A*
C1—H1*B*⋯O1^i^	0.98	2.59	3.469 (7)	150
C6—H6⋯Cl1^ii^	0.95	2.77	3.457 (6)	130
C4—H4⋯O2^iii^	0.95	2.62	3.563 (5)	171

Symmetry codes: (i) 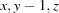; (ii) 
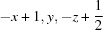
; (iii) 
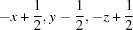
.

## supplementary crystallographic information

### S1. Comment

Tolylfluanid, C_10_H_13_Cl_2_FN_2_O_2_S_2_, is a member of the phenylsulfamide group of fungicides and has been applied in agriculture for control of fungal diseases during thinning, pruning, and harvesting of fruits and vegetables (Sargis *et al.*, 2012; Stajnbaher & Zupancic-Kralj, 2008). Its crystal structure is reported herein. In this compound (Scheme 1, Fig. 1), the dihedral angle between the dimethylamino group plane and that of the phenyl ring is 32.3 (3)°. Disorder was modeled for one Cl atom (Cl1) and one F atom (F1) of the dichlorofluoromethylthio group over two sets of sites with an occupancy ratio of 0.605 (9):0.395 (9). All bond lengths and bond angles are normal and comparable to those observed in the crystal structure of a similar compound (Ogata *et al.*, 1986).

In the crystal structure, two C6–H6..Cl1 hydrogen bonds link adjacent molecules, forming dimers with *R*_2_^2^(14) loops. C1–H1B···O1 hydrogen bonds link pairs of dimers into chains along the *b* axis direction. These chains are joined by an additional slightly weaker C4–H4···O2 contact generating a two-dimensional sheet in the *ab* plane.

### S2. Experimental

The title compound was purchased from the Dr. Ehrenstorfer GmbH Company. Slow evaporation from a solution in CHCl_3_ gave single crystals suitable for X-ray analysis.

### S3. Refinement

All H-atoms were positioned geometrically and refined using a riding model with d(C—H) = 0.98 Å, *U*_iso_ = 1.5*U*_eq_(C) for methyl group and d(C—H) = 0.95 Å, *U*_iso_ = 1.2*U*_eq_(C) for C*sp*^2^—H.

### Crystal data

**Table:**

C_10_H_13_Cl_2_FN_2_O_2_S_2_	*F*(000) = 1424
*M**_r_* = 347.24	*D*_x_ = 1.556 Mg m^−^^3^
Monoclinic, *C*2/*c*	Mo *K*α radiation, λ = 0.71073 Å
Hall symbol: -C 2yc	Cell parameters from 6794 reflections
*a* = 23.7638 (19) Å	θ = 2.8–27.1°
*b* = 8.7046 (7) Å	µ = 0.73 mm^−^^1^
*c* = 14.6559 (11) Å	*T* = 173 K
β = 102.133 (3)°	Block, colourless
*V* = 2963.9 (4) Å^3^	0.19 × 0.10 × 0.08 mm
*Z* = 8	

### Data collection

**Table:**

Bruker APEXII CCD diffractometer	2913 independent reflections
Radiation source: fine-focus sealed tube	2236 reflections with *I* > 2σ(*I*)
Graphite monochromator	*R*_int_ = 0.059
φ and ω scans	θ_max_ = 26.0°, θ_min_ = 2.5°
Absorption correction: multi-scan (*SADABS*; Bruker, 2009)	*h* = −29→26
*T*_min_ = 0.874, *T*_max_ = 0.944	*k* = −10→10
18246 measured reflections	*l* = −18→18

### Refinement

**Table:**

Refinement on *F*^2^	Primary atom site location: structure-invariant direct methods
Least-squares matrix: full	Secondary atom site location: difference Fourier map
*R*[*F*^2^ > 2σ(*F*^2^)] = 0.058	Hydrogen site location: inferred from neighbouring sites
*wR*(*F*^2^) = 0.135	H-atom parameters constrained
*S* = 1.14	*w* = 1/[σ^2^(*F*_o_^2^) + (0.0259*P*)^2^ + 15.3569*P*] where *P* = (*F*_o_^2^ + 2*F*_c_^2^)/3
2913 reflections	(Δ/σ)_max_ < 0.001
194 parameters	Δρ_max_ = 0.48 e Å^−^^3^
0 restraints	Δρ_min_ = −0.50 e Å^−^^3^

### Special details

**Table:**

Geometry. All e.s.d.'s (except the e.s.d. in the dihedral angle between two l.s. planes) are estimated using the full covariance matrix. The cell e.s.d.'s are taken into account individually in the estimation of e.s.d.'s in distances, angles and torsion angles; correlations between e.s.d.'s in cell parameters are only used when they are defined by crystal symmetry. An approximate (isotropic) treatment of cell e.s.d.'s is used for estimating e.s.d.'s involving l.s. planes.
Refinement. Refinement of *F*^2^ against ALL reflections. The weighted *R*-factor *wR* and goodness of fit *S* are based on *F*^2^, conventional *R*-factors *R* are based on *F*, with *F* set to zero for negative *F*^2^. The threshold expression of *F*^2^ > σ(*F*^2^) is used only for calculating *R*-factors(gt) *etc*. and is not relevant to the choice of reflections for refinement. *R*-factors based on *F*^2^ are statistically about twice as large as those based on *F*, and *R*- factors based on ALL data will be even larger.

### Fractional atomic coordinates and isotropic or equivalent isotropic displacement parameters (Å^2^)

**Table:**

	*x*	*y*	*z*	*U*_iso_*/*U*_eq_	Occ. (<1)
Cl1	0.42965 (16)	0.2602 (5)	0.1267 (3)	0.0592 (11)	0.605 (9)
Cl1'	0.3886 (3)	0.4591 (10)	0.1620 (5)	0.0549 (18)	0.395 (9)
Cl2	0.32595 (6)	0.33538 (16)	−0.00564 (8)	0.0545 (4)	
S1	0.33566 (4)	0.30575 (13)	0.35656 (7)	0.0302 (3)	
S2	0.31078 (4)	0.20191 (13)	0.16220 (7)	0.0307 (3)	
F1	0.3625 (4)	0.4708 (12)	0.1530 (7)	0.052 (2)	0.605 (9)
F1'	0.4106 (7)	0.1951 (19)	0.1104 (9)	0.066 (4)	0.395 (9)
O1	0.38994 (13)	0.3659 (4)	0.4004 (2)	0.0438 (8)	
O2	0.29116 (13)	0.4060 (4)	0.31161 (19)	0.0402 (8)	
N1	0.34588 (14)	0.1819 (4)	0.2728 (2)	0.0304 (8)	
N2	0.31214 (16)	0.2043 (4)	0.4323 (2)	0.0376 (9)	
C1	0.4891 (2)	−0.3490 (7)	0.3831 (4)	0.0685 (17)	
H1A	0.5214	−0.3487	0.3510	0.103*	
H1B	0.4661	−0.4421	0.3663	0.103*	
H1C	0.5040	−0.3467	0.4507	0.103*	
C2	0.4519 (2)	−0.2090 (6)	0.3541 (3)	0.0428 (11)	
C3	0.39365 (19)	−0.2240 (6)	0.3152 (3)	0.0385 (11)	
H3	0.3771	−0.3236	0.3064	0.046*	
C4	0.35902 (18)	−0.0958 (5)	0.2887 (3)	0.0334 (10)	
H4	0.3192	−0.1084	0.2623	0.040*	
C5	0.38234 (17)	0.0485 (5)	0.3007 (3)	0.0308 (9)	
C6	0.4402 (2)	0.0684 (6)	0.3386 (4)	0.0474 (12)	
H6	0.4565	0.1684	0.3458	0.057*	
C7	0.4738 (2)	−0.0593 (6)	0.3658 (4)	0.0585 (15)	
H7	0.5133	−0.0452	0.3937	0.070*	
C8	0.2585 (2)	0.1181 (6)	0.3994 (4)	0.0546 (14)	
H8A	0.2674	0.0187	0.3743	0.082*	
H8B	0.2331	0.1768	0.3503	0.082*	
H8C	0.2393	0.1014	0.4515	0.082*	
C9	0.3532 (3)	0.1351 (7)	0.5110 (3)	0.0612 (16)	
H9A	0.3347	0.1233	0.5644	0.092*	
H9B	0.3870	0.2018	0.5285	0.092*	
H9C	0.3652	0.0341	0.4925	0.092*	
C10	0.3611 (2)	0.3152 (6)	0.1127 (3)	0.0458 (12)	

### Atomic displacement parameters (Å^2^)

**Table:**

	*U*^11^	*U*^22^	*U*^33^	*U*^12^	*U*^13^	*U*^23^
Cl1	0.0428 (17)	0.057 (2)	0.084 (2)	−0.0029 (13)	0.0277 (15)	0.0112 (17)
Cl1'	0.070 (4)	0.052 (3)	0.048 (2)	−0.015 (3)	0.025 (3)	−0.0026 (19)
Cl2	0.0701 (9)	0.0687 (9)	0.0275 (6)	0.0036 (7)	0.0163 (6)	0.0102 (6)
S1	0.0345 (6)	0.0318 (6)	0.0237 (5)	−0.0008 (5)	0.0050 (4)	−0.0004 (4)
S2	0.0316 (6)	0.0378 (6)	0.0221 (5)	0.0014 (5)	0.0044 (4)	0.0015 (4)
F1	0.065 (5)	0.038 (4)	0.056 (4)	−0.010 (5)	0.025 (5)	−0.009 (3)
F1'	0.066 (8)	0.079 (10)	0.066 (7)	0.057 (7)	0.047 (7)	0.028 (7)
O1	0.0412 (19)	0.044 (2)	0.0430 (18)	−0.0107 (15)	0.0016 (14)	−0.0031 (15)
O2	0.0482 (19)	0.0385 (19)	0.0328 (16)	0.0149 (15)	0.0062 (14)	−0.0027 (14)
N1	0.0344 (19)	0.036 (2)	0.0203 (16)	0.0061 (16)	0.0038 (14)	−0.0011 (15)
N2	0.050 (2)	0.040 (2)	0.0251 (17)	−0.0075 (18)	0.0119 (16)	−0.0017 (16)
C1	0.058 (4)	0.050 (4)	0.091 (4)	0.011 (3)	−0.001 (3)	0.024 (3)
C2	0.041 (3)	0.043 (3)	0.045 (3)	0.003 (2)	0.010 (2)	0.009 (2)
C3	0.044 (3)	0.038 (3)	0.035 (2)	−0.001 (2)	0.010 (2)	0.000 (2)
C4	0.029 (2)	0.043 (3)	0.027 (2)	0.0004 (19)	0.0039 (17)	−0.0059 (19)
C5	0.032 (2)	0.036 (3)	0.025 (2)	0.0080 (19)	0.0069 (17)	0.0035 (18)
C6	0.034 (3)	0.035 (3)	0.069 (3)	−0.004 (2)	0.003 (2)	0.010 (2)
C7	0.034 (3)	0.050 (3)	0.083 (4)	0.000 (2)	−0.007 (3)	0.014 (3)
C8	0.061 (3)	0.052 (3)	0.062 (3)	−0.019 (3)	0.038 (3)	−0.013 (3)
C9	0.089 (4)	0.060 (4)	0.033 (3)	0.007 (3)	0.010 (3)	0.019 (3)
C10	0.044 (3)	0.062 (4)	0.033 (2)	−0.002 (2)	0.011 (2)	0.010 (2)

### Geometric parameters (Å, º)

**Table:**

Cl1—C10	1.670 (6)	C1—H1C	0.9800
Cl1'—C10	1.523 (10)	C2—C3	1.389 (6)
Cl2—C10	1.768 (5)	C2—C7	1.400 (7)
S1—O1	1.415 (3)	C3—C4	1.392 (6)
S1—O2	1.422 (3)	C3—H3	0.9500
S1—N2	1.608 (3)	C4—C5	1.369 (6)
S1—N1	1.690 (3)	C4—H4	0.9500
S2—N1	1.669 (3)	C5—C6	1.381 (6)
S2—C10	1.814 (5)	C6—C7	1.378 (7)
F1—C10	1.474 (12)	C6—H6	0.9500
F1'—C10	1.581 (11)	C7—H7	0.9500
N1—C5	1.455 (5)	C8—H8A	0.9800
N2—C8	1.470 (6)	C8—H8B	0.9800
N2—C9	1.473 (6)	C8—H8C	0.9800
C1—C2	1.513 (7)	C9—H9A	0.9800
C1—H1A	0.9800	C9—H9B	0.9800
C1—H1B	0.9800	C9—H9C	0.9800
			
O1—S1—O2	120.1 (2)	C7—C6—H6	120.6
O1—S1—N2	107.78 (19)	C5—C6—H6	120.6
O2—S1—N2	108.94 (19)	C6—C7—C2	122.5 (5)
O1—S1—N1	108.03 (18)	C6—C7—H7	118.8
O2—S1—N1	105.17 (17)	C2—C7—H7	118.8
N2—S1—N1	105.96 (18)	N2—C8—H8A	109.5
N1—S2—C10	102.03 (19)	N2—C8—H8B	109.5
C5—N1—S2	120.2 (3)	H8A—C8—H8B	109.5
C5—N1—S1	118.3 (2)	N2—C8—H8C	109.5
S2—N1—S1	121.3 (2)	H8A—C8—H8C	109.5
C8—N2—C9	115.7 (4)	H8B—C8—H8C	109.5
C8—N2—S1	117.2 (3)	N2—C9—H9A	109.5
C9—N2—S1	119.7 (3)	N2—C9—H9B	109.5
C2—C1—H1A	109.5	H9A—C9—H9B	109.5
C2—C1—H1B	109.5	N2—C9—H9C	109.5
H1A—C1—H1B	109.5	H9A—C9—H9C	109.5
C2—C1—H1C	109.5	H9B—C9—H9C	109.5
H1A—C1—H1C	109.5	F1—C10—Cl1'	23.6 (3)
H1B—C1—H1C	109.5	F1—C10—F1'	131.4 (7)
C3—C2—C7	116.8 (4)	Cl1'—C10—F1'	107.8 (7)
C3—C2—C1	120.9 (5)	F1—C10—Cl1	106.0 (5)
C7—C2—C1	122.4 (4)	Cl1'—C10—Cl1	82.3 (4)
C2—C3—C4	121.3 (4)	F1'—C10—Cl1	25.9 (7)
C2—C3—H3	119.4	F1—C10—Cl2	105.5 (5)
C4—C3—H3	119.4	Cl1'—C10—Cl2	116.8 (4)
C5—C4—C3	120.0 (4)	F1'—C10—Cl2	104.1 (6)
C5—C4—H4	120.0	Cl1—C10—Cl2	113.3 (3)
C3—C4—H4	120.0	F1—C10—S2	107.5 (5)
C4—C5—C6	120.6 (4)	Cl1'—C10—S2	120.7 (4)
C4—C5—N1	119.7 (4)	F1'—C10—S2	101.9 (7)
C6—C5—N1	119.8 (4)	Cl1—C10—S2	120.2 (3)
C7—C6—C5	118.9 (4)	Cl2—C10—S2	103.4 (2)
			
C10—S2—N1—C5	−92.6 (3)	C3—C4—C5—C6	0.2 (6)
C10—S2—N1—S1	92.9 (3)	C3—C4—C5—N1	180.0 (4)
O1—S1—N1—C5	60.6 (3)	S2—N1—C5—C4	−59.3 (5)
O2—S1—N1—C5	−170.0 (3)	S1—N1—C5—C4	115.4 (4)
N2—S1—N1—C5	−54.7 (3)	S2—N1—C5—C6	120.5 (4)
O1—S1—N1—S2	−124.8 (2)	S1—N1—C5—C6	−64.9 (5)
O2—S1—N1—S2	4.6 (3)	C4—C5—C6—C7	−1.2 (7)
N2—S1—N1—S2	119.9 (2)	N1—C5—C6—C7	179.0 (4)
O1—S1—N2—C8	−174.3 (3)	C5—C6—C7—C2	1.9 (8)
O2—S1—N2—C8	53.9 (4)	C3—C2—C7—C6	−1.5 (8)
N1—S1—N2—C8	−58.8 (4)	C1—C2—C7—C6	179.3 (5)
O1—S1—N2—C9	−25.5 (4)	N1—S2—C10—F1	−69.9 (5)
O2—S1—N2—C9	−157.4 (4)	N1—S2—C10—Cl1'	−48.4 (5)
N1—S1—N2—C9	89.9 (4)	N1—S2—C10—F1'	70.9 (7)
C7—C2—C3—C4	0.4 (7)	N1—S2—C10—Cl1	51.2 (4)
C1—C2—C3—C4	179.7 (5)	N1—S2—C10—Cl2	178.7 (2)
C2—C3—C4—C5	0.2 (6)		

### Hydrogen-bond geometry (Å, º)

**Table:**

*D*—H···*A*	*D*—H	H···*A*	*D*···*A*	*D*—H···*A*
C1—H1*B*···O1^i^	0.98	2.59	3.469 (7)	150
C6—H6···Cl1^ii^	0.95	2.77	3.457 (6)	130
C4—H4···O2^iii^	0.95	2.62	3.563 (5)	171

Symmetry codes: (i) *x*, *y*−1, *z*; (ii) −*x*+1, *y*, −*z*+1/2; (iii) −*x*+1/2, *y*−1/2, −*z*+1/2.

## References

[bb1] Brandenburg, K. (2010). *DIAMOND* Crystal Impact GbR, Bonn, Germany.

[bb2] Bruker (2009). *APEX2*, *SAINT* and *SADABS* Bruker AXS Inc., Madison, Wisconsin, USA.

[bb3] Ogata, M., Matsumoto, H., Shimizu, S., Kida, S., Wada, T., Shiro, M. & Sato, K. (1986). *J. Med. Chem.* **29**, 417–423. 10.1021/jm00153a018 3005577

[bb4] Sargis, R. M., Neel, B. A., Brock, C. O., Lin, Y., Hickey, A. T., Carlton, D. A. & Brady, M. J. (2012). *Biochim. Biophys. Acta*, **1822**, 952–960. 10.1016/j.bbadis.2012.02.015 PMC3338892 22387882

[bb5] Sheldrick, G. M. (2008). *Acta Cryst.* A**64**, 112–122. 10.1107/S0108767307043930 18156677

[bb6] Stajnbaher, D. & Zupancic-Kralj, L. (2008). *J. Chromatogr.* **1190**, 316–326. 10.1016/j.chroma.2008.03.002 18367194

