# Supplementary figures and images for: Crystal structure of tolyl­fluanid
Source: Acta Crystallogr Sect E Struct Rep Online. 2014 Sep 20;70(Pt 10):o1114–5. doi: 10.1107/S1600536814020741 (PMC4257202; doi:10.1107/S1600536814020741)

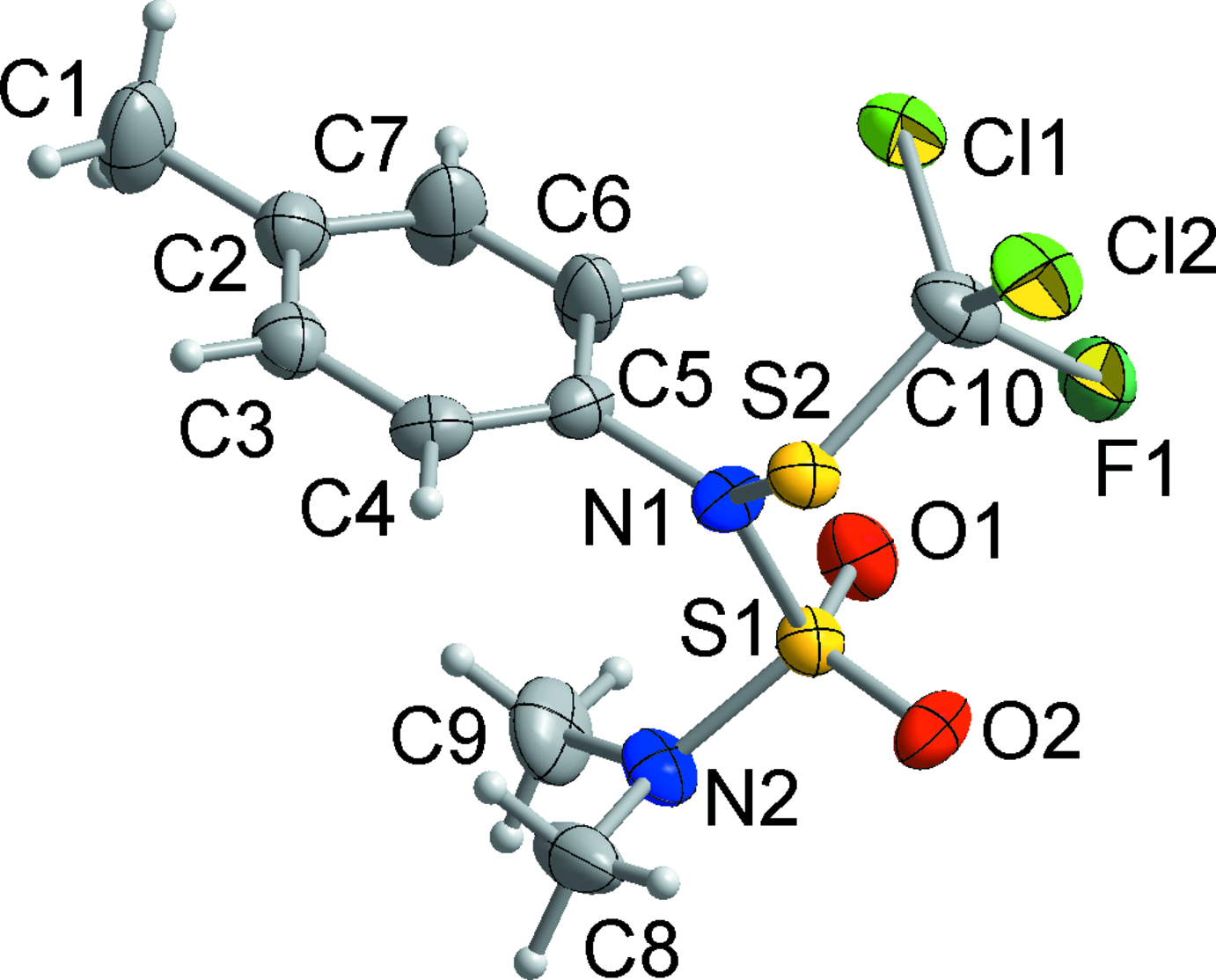

Supplement: Supplementary file 3 [file e-70-o1114-fig1.tif]

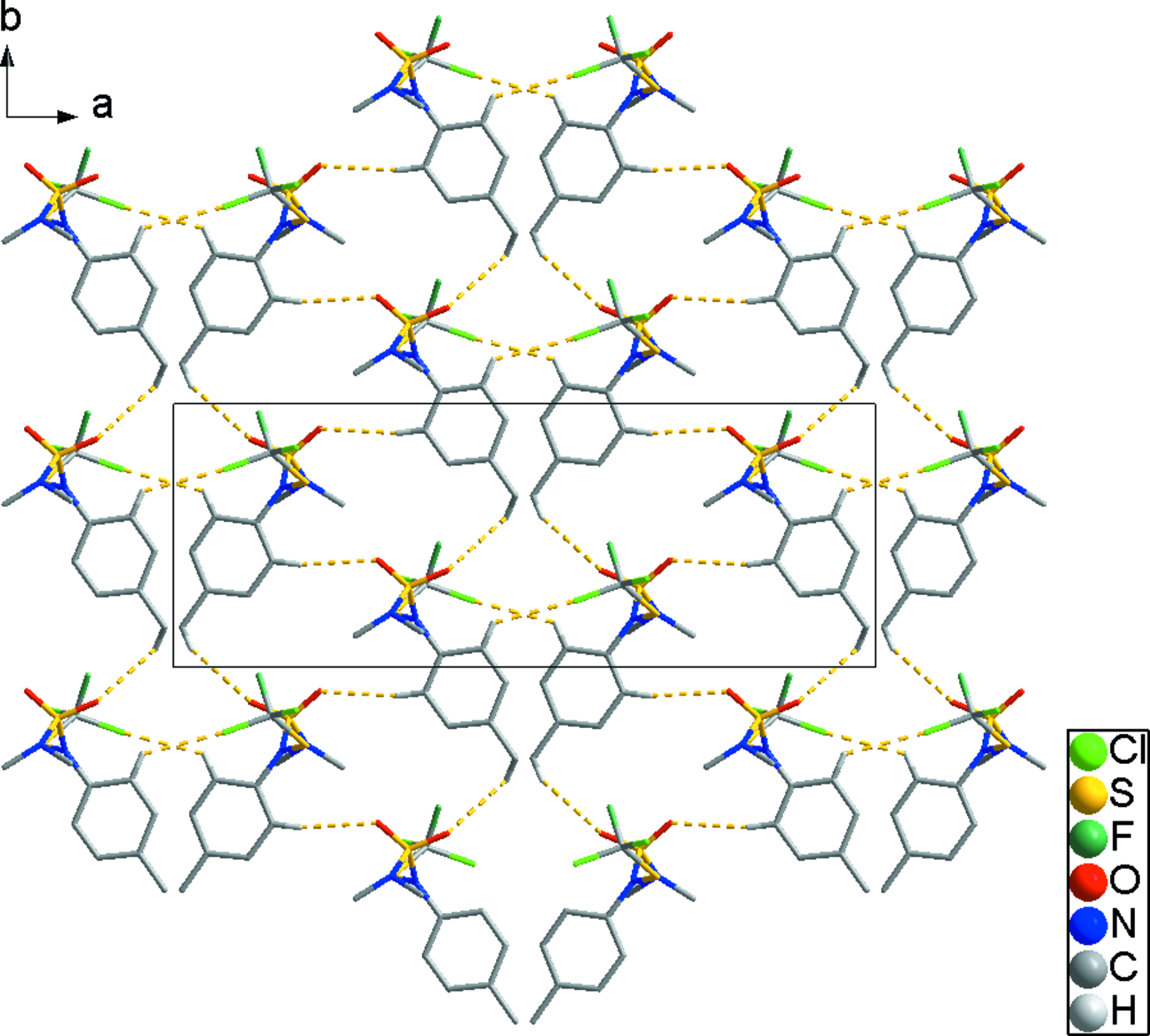

Supplement: Supplementary file 4 [file e-70-o1114-fig2.tif]
